# Is Emergency Surgery an Indicator of Good Functional Outcomes in Neck of Femur Fractures Among Adults: A Prospective Clinical Study

**DOI:** 10.7759/cureus.23074

**Published:** 2022-03-11

**Authors:** Abhi Sharma, Arun H Shanthappa, Sandesh Agarawal

**Affiliations:** 1 Orthopaedics, Sri Devaraj Urs Academy of Higher Education and Research, Kolar, IND

**Keywords:** india, functional assessment, complications, timing of the surgery, fracture neck of femur

## Abstract

Background

Because of their high frequency and severity, femoral neck fractures are a major public health concern. There is a scarcity of recorded literature that relates the timing of surgery, the effect of displacement, and the tamponade effect of the neck of the femur fracture (FNF) issues such as non-union and avascular necrosis of the femoral head after surgery. This study aimed to assess the association between the timing of the surgery and its functional outcome and surgical complications.

Methodology

This observational study was done among patients diagnosed with an FNF who were admitted in a tertiary care center for a period of three years. The study included 36 participants who were selected using the universal sampling technique. Regarding the timing of the surgery, the study participants were divided into two groups, namely, patients who were operated on before 24 hours and those who were operated on after 24 hours.

Results

The mean age of the study participants was 36.19 years, and about 75% were males. About 25% of the participants had a complication. There was a statistically significant association between functional assessment at 24 months and surgery done before 24 hours and non-displaced fracture. Moreover, there was a statistically significant association between the timing of surgery before 24 hours and fewer complications.

Conclusions

In young adults, the timing of the surgery (before 24 hours) had good functional outcomes after 24 months than the late timing of the surgery (after 24 hours). Whereas there was no statistically significant difference for functional outcomes at the one-month follow-up with the timing of the surgery. Males had a high probability of getting an FNF. The prevalence of complications was low in individuals operated on before 24 hours.

## Introduction

Every year, about 4.5 million people become crippled as a result of a hip fracture, with the number of people living with a hip fracture disability predicted to rise to 21 million in the next 40 years. Despite surgical intervention, the need for reoperation is still considerable (10.0-48.8%), has remained virtually stable over the last 30 years, and is linked to significant morbidity and expenditures [1-3]. Because femoral neck fractures have a high frequency and severity, they are a foremost public health concern. These life-threatening occurrences can affect limb function and have a one-year death incidence of 17-24%. Half of all femoral neck fractures are displaced intracapsular fractures that jeopardize the femoral head’s blood supply and, as a result, the odds of bone repair [4]. These fractures cause significant damage to the extracapsular arterial ring’s ascending cervical arteries. The nutrition of the head of the femur rest on the degree of injury and how much the retinal arteries are involved [5]. Because femoral neck fractures are surgically treated and related to significant mortality and morbidity, it is critical to adopt effective and efficient treatment procedures that take into account factors such as age, fracture complexity, and the presence of concomitant injuries [6]. Avascular necrosis of the femoral head and non-union have been observed in multiple studies following surgical treatment of femoral neck fractures [7-10]. Several other aspects have also been studied in these fractures, including vascular damage, tamponade effect, fracture displacement, surgical treatment delay, and surgical procedures [11]. The objective of surgical treatment in these fractures should be to achieve adequate anatomic reduction and stable fixation. Moreover, the functional outcomes of the patients after surgery have implications in reducing the disability among the injured and help in regaining the optimal maximum health. Additionally, it helps in reducing the economic burden experienced by the individual and the society in terms of rehabilitation of the injured. There is a scarcity of recorded literature that relates the timing of surgery and the effect of displacement with functional outcomes after the surgery [12,13]. With this background, this study aimed to assess the association between the timing of the surgery and its functional outcomes and surgical complications in adult patients.

## Materials and methods

This study was an observational prospective study done among patients diagnosed with a neck of the femur fracture (FNF) who were admitted to R.L. Jalappa Hospital Centre for a period of three years (from January 2016 to January 2019). Ethical clearance was obtained from the Institutional Ethical Committee of R.L. Jalappa Hospital Centre. During the study period, 38 eligible patients were included in the study, of whom two were not willing to participate. Thus, this study was conducted among 36 study participants who were selected using the universal sampling technique. Patients who were willing to participate in the study were aged 18 to 50 years. Patients who underwent closed reduction technique and cannulated cancellous screw fixation were included in this study. Patients who were diagnosed with pathological fractures and fractures associated with neurovascular injuries were excluded from the study. Regarding the timing of the surgery, the study participants were divided into two groups, namely, patients who were operated on before 24 hours and those who were operated on after 24 hours. The fracture was classified using Garden Classification, which is based on radiological appearance. Individuals with an incomplete fracture line or an impact fracture were grouped as type I, whereas those with a full but non-displaced fracture were grouped as type II. Type III cases had a complete fracture and displacement rate of less than 50%, whereas type IV cases had a full fracture and displacement rate of more than 50% [14]. Non-union was defined as insufficient fixation, loss of reduction, or a detectable fracture line at 12 months.

The Ficat criteria were used to assess avascular necrosis in the femoral head [15]. Harris Hip Scores were used to assess the functional results of the hip [16]. The FNF cases were operated on the traction table under spinal anesthesia. Under fluoroscopic direction, a closed reduction was achieved. In a longitudinal and equilateral triangle position, three half-grooved cannulated screws with a diameter of 6.5 mm were used. All patients were administered 1 g ceftriaxone prophylactically one hour before surgery and twice daily for 48 hours. On postoperative day two, patients were started on non-weight-bearing mobility with walker support. When evidence of callus formation was seen on the follow-up X-ray, patients were allowed partial weight-bearing mobilization after 12 weeks. Clinical information was gathered and evaluated using the Harris Hip Score and the Ficat Arlet score. The information gathered was recorded into a Microsoft Excel spreadsheet and analyzed using SPSS version 21 (IBM Corp., Armonk, NY, USA). The chi-square/Fisher’s exact test was used to calculate the relationship between qualitative data in the form of a bar diagram. For quantitative data, the association was calculated using an independent t-test and expressed as mean and standard deviation.

## Results

In our study, 36 individuals participated. The mean age of the study participants was 36.19 years, as shown in Table 1. The baseline features of the study participants are shown in Table 2.

**Table 1:** Age distribution of the study participants (n = 36).

Age (in years)	
Mean	36.19
Standard deviation	6.828
Minimum	20
Maximum	50

**Table 2:** Baseline features of the study participants (n = 36).

Baseline characteristics of the study participants		Frequency	Percentage
Gender	Female	9	25.0
	Male	27	75.0
Affected side	Left	16	44.4
	Right	20	55.6
Timing of surgery before 24 hours	No	20	55.6
	Yes	16	44.4
Type of fracture	Non-displaced fracture	22	61.1
	Displaced fracture	14	38.9
Complications	No	27	75.0
	Yes	9	25.0
Complication details	Avascular necrosis	3	8.3
	Nil	29	80.6
	Non-union	4	11.1
Classification	Type 1	8	22.2
	Type 2	14	38.9
	Type 3	9	25.0
	Type 4	5	13.9

The functional assessment scores (Harris Hip Scores) of the study participants during the follow-up period are shown in Tables 3, 4.

**Table 3:** Distribution of study participants according to the functional assessment scores during follow-up (n = 36).

		Functional assessment at one month	Functional assessment at two months	Functional assessment at six months	Functional assessment at 12 months	Functional assessment at 24 months
Mean		54.42	68.47	73.72	76.00	79.03
Median		55.00	67.00	75.00	76.00	80.00
Mode		45	65	75	75	75
Standard deviation		8.680	5.759	4.213	4.733	6.780
Minimum		43	60	65	65	64
Maximum		78	78	80	84	90
Percentiles	25	45.25	65.00	70.00	75.00	75.00
	50	55.00	67.00	75.00	80.00	80.00
	75	58.75	73.75	77.00	84.75	85.00

**Table 4:** Distribution of study participants according to the functional assessment score categories during follow-up (n = 36).

		Frequency	Percentage
Functional assessment at one month	Fair	1	2.8
	Poor	35	97.2
Functional assessment at two months	Fair	15	41.7
	Poor	21	58.3
Functional assessment at six months	Fair	27	75.0
	Good	3	8.3
	Poor	6	16.7
Functional assessment at 12 months	Fair	24	66.7
	Good	9	25.0
	Poor	3	8.3
Functional assessment at 24 months	Excellent	2	5.6
	Fair	12	33.3
	Good	17	47.2
	Poor	5	13.9

**Figure 1:**
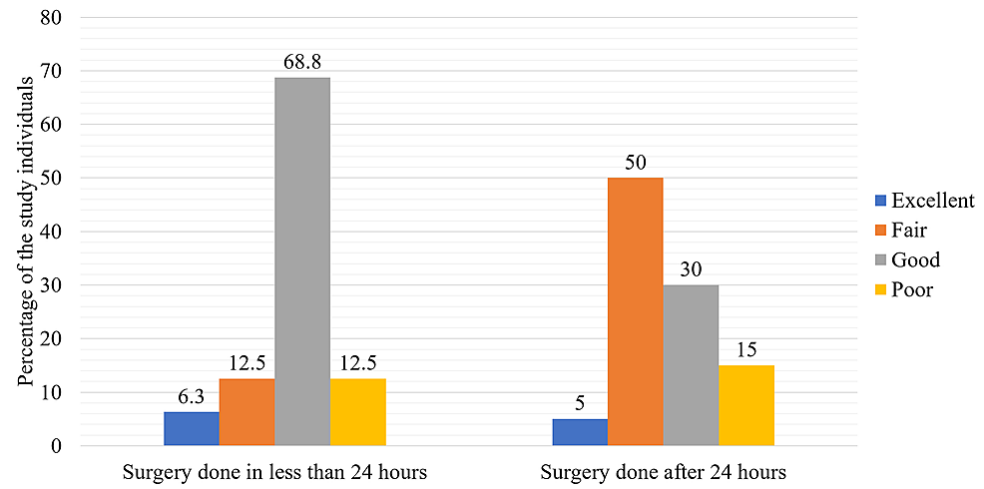
Distribution of study participants according to the functional assessment score categories at 24 months with respect to the timing of the surgery (n = 36).

**Figure 2:**
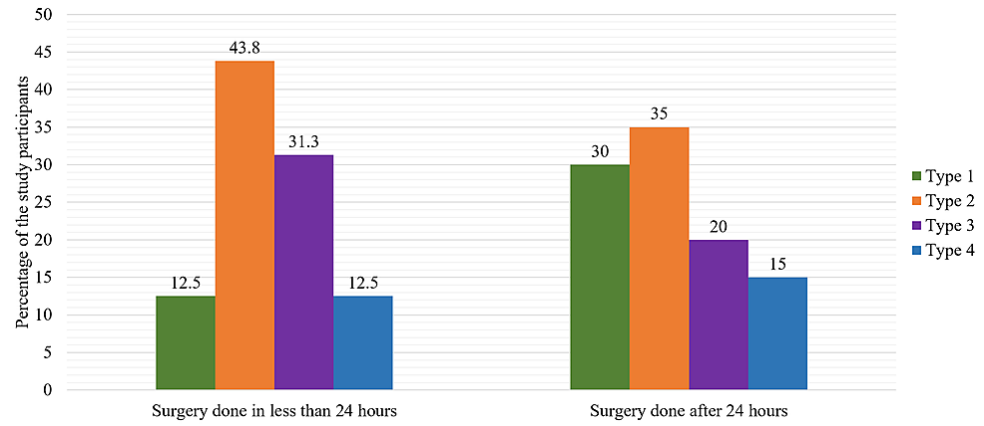
Distribution of study participants according to the classification of the fracture with respect to the timing of the surgery (n = 36).

The study participants are described in terms of two groups for functional assessment at 24 months, classification of fracture, and complications in Figures 1, 2 and Table 5.

**Table 5:** Distribution of study participants according to the complications with respect to the timing of the surgery (n = 36).

Timing of surgery	Complication	Frequency	Percentage
Surgery done in less than 24 hours	Avascular necrosis	1	6.3
	Nil	14	87.5
	Non-union	1	6.3
Surgery done after 24 hours	Avascular necrosis	2	10.0
	Nil	15	75.0
	Non-union	3	15.0

According to the independent t-test, there was a statistically significant difference in the mean difference between the two groups (surgery done in less than 24 hours and surgery done after 24 hours) with respect to functional assessment at 24 months. Surgery done in less than 24 hours had better functionality, as shown in Table 6.

**Table 6:** Association of functional assessment at one and 24 months with the timing of the surgery (n = 36).

	Timing of surgery	Mean	Standard deviation	Mean difference	P-value
Functional assessment at one month	Surgery done in less than 24 hours	53.06	8.394	-2.438	0.410
	Surgery done after 24 hours	55.50	8.965		
Functional assessment at 24 months	Surgery done in less than 24 hours	82.38	5.315	6.025	0.006
	Surgery done after 24 hours	76.35	6.738		

According to the independent t-test, there was a statistically significant difference between both groups (non-displaced fracture and displaced fracture) with respect to functional assessment at 24 months. Non-displaced fractures had better functionality, as shown in Table 7.

**Table 7:** Association of functional assessment 12 months with the type of fracture (n = 36).

	Type of fracture	Mean	Standard deviation	Mean difference	P-value
Functional assessment at 24 months	Non-displaced fracture	81.45	6.155	6.2402	0.004
	Displaced fracture	75.21	6.079		

Of the study participants who underwent surgery before 24 hours, 93.8% had no complications compared to 60% among individuals who underwent surgery after 24 hours. This difference in proportion was statistically significant (p = 0.026) using the chi-square test, as shown in Table 8.

**Table 8:** Association of complications with the timing of the surgery (n = 36).

			Complications		Total	Chi-square value	P-value
			No	Yes			
Timing of surgery before 24 hours	No	Count	12	8	20	3.750	0.026
		%	60.0%	40.0%	100.0%		
	Yes	Count	15	1	16		
		%	93.8%	6.3%	100.0%		
Timing of surgery after 24 hours	No	Count	14	1	15	3.086	0.032
		%	93.3%	6.7%	100.0%		
	Yes	Count	13	8	21		
		%	61.9%	38.1%	100.0%		

## Discussion

The goal of this study was to determine if factors such as surgical timing and displacement affected the rate of complications and functional scores in patients with FNF. According to the literature, the goal of therapy in young people with femoral neck fractures is to offer fracture healing, prohibit osteonecrosis by maintaining the femur head, and provide quick rehabilitation to allow patients to regain their health [17,18]. Our study showed that the mean age of the study participants was 36.19 years, and about 75% of the study participants were males. About 25% of the individuals had complications, with non-union being the most common complication. Regarding avascular necrosis, our study showed a prevalence of 8.3%. In contrast to our study, a study by Mao et al., in China, among 212 cases found a prevalence of 33% for AVN [19]. Another study done by Slobogean et al. in Canada, among 1,558 individuals, found that the prevalence of AVN among isolated FNF cases was 14.3% [20]. This difference could be due to the difference in the surgical procedure and the study area. Our study reported no mortality. In contrast to this study, a study by Sheikh et al., in London, showed that the mortality among patients who underwent surgery after FNF was 8.7% [21]. This difference could be due to the difference in the sample size. A multicentric study with a large sample yields better results.

Our study showed a statistically significant association between functional assessment at 24 months and surgery done before 24 hours as well as between functional assessment at 24 months and non-displaced fracture. Additionally, our study showed a statistically significant association between the timing of surgery before 24 hours and fewer complications. Similar to our study, a study done by Smektala et al., in Germany, among 2,916 hip fracture patients, concluded that there was an association between less time to surgery and complications, but there was no statistically significant association between less time to surgery and mortality [22]. Similar to our study, a study by Popelka et al. among 58 femoral neck fracture patients who were surgically treated found that the patients who underwent surgery before 24 hours had a statistically significant lower rate of developing avascular necrosis [23]. In contrast to the above discussion, a study by Upadhayay et al., in New Delhi, among 102 patients of FNF, found that there was no association between the timing of the surgery (after 48 hours) and complications (union and avascular necrosis) [24]. Another study by Araujo et al., in Brazil, among 31 patients diagnosed with displaced FNF, found that there was no statistically significant difference between the timing of the surgery and complications [25]. Thus, this topic needs further investigation in various settings.

Strengths and limitations

Because the data collected were recorded data, the reliability was verified thoroughly. The data collection and data entry were done by a single investigator which avoided bias in this study. Although this study employed universal sampling, more samples can yield better results. A multicentric study design could be a better choice. This study yields various associations that can be studied in controlled trials in various settings that will provide further evidence.

## Conclusions

In young adults, the timing of the surgery done before 24 hours had good functional outcomes after 24 months than the late timing of the surgery (after 24 hours). However, there was no statistically significant difference for functional outcomes at the one-month follow-up with the timing of the surgery. Among young adults, males have a high probability of an FNF. The prevalence of complications was low in individuals who were operated on before 24 hours than those who were operated on late. Thus, when it comes to the management of FNF among young adults, the timing of the surgery (before 24 hours) is important in deciding the functional outcomes of the patients, which further influences the individual disability.
